# Age-Specific Activation Patterns and Inter-Subject Similarity During Verbal Working Memory Maintenance and Cognitive Reserve

**DOI:** 10.3389/fpsyg.2022.852995

**Published:** 2022-06-09

**Authors:** Christian Habeck, Yunglin Gazes, Yaakov Stern

**Affiliations:** Cognitive Neuroscience Division, Department of Neurology, Columbia University Irving Medical Center, New York, NY, United States

**Keywords:** cognitive reserve, fMRI, multivariate analysis, verbal working memory, inter-subject similarity

## Abstract

Cognitive Reserve (CR), according to a recent consensus definition of the NIH-funded Reserve and Resilience collaboratory,^1^ is constituted by any mechanism contributing to cognitive performance beyond, or interacting with, brain structure in the widest sense. To identity multivariate activation patterns fulfilling this postulate, we investigated a verbal Sternberg fMRI task and imaged 181 people with age coverage in the ranges 20–30 (44 participants) and 55–70 (137 participants). Beyond task performance, participants were characterized in terms of demographics, and neuropsychological assessments of vocabulary, episodic memory, perceptual speed, and abstract fluid reasoning. Participants studied an array of either one, three, or six upper-case letters for 3 s (=encoding phase), then a blank fixation screen was presented for 7 s (=maintenance phase), to be probed with a lower-case letter to which they responded with a differential button press whether the letter was part of the studied array or not (=retrieval phase). We focused on identifying maintenance-related activation patterns showing memory load increases in pattern score on an individual participant level for both age groups. We found such a pattern that increased with memory load for all but one person in the young participants (*p* < 0.001), and such a pattern for all participants in the older group (*p* < 0.001). Both patterns showed broad topographic similarities; however, relationships to task performance and neuropsychological characteristics were markedly different and point to individual differences in Cognitive Reserve. Beyond the derivation of group-level activation patterns, we also investigated the inter-subject spatial similarity of individual working memory rehearsal patterns in the older participants’ group as a function of neuropsychological and task performance, education, and mean cortical thickness. Higher task accuracy and neuropsychological function was reliably associated with higher inter-subject similarity of individual-level activation patterns in older participants.

## Introduction

The objective of the current study was to use the well-studied letter Working-Memory Sternberg task (Braver et al., 1997; Cohen et al., 1997; Jonides et al., 1997; Manoach et al., 1997; Smith and Jonides, 1997, 1998; Smith et al., 1998; D'Esposito et al., 1999, 2000; Postle and D’Esposito, 1999; Rypma et al., 1999, 2001, 2002; Rypma and D’Esposito, 1999, 2000, 2003; Postle et al., 2000a,b) to investigate the associated activation patterns as possible mechanism of Cognitive Reserve (CR). Cognitive Reserve and its contributions to, and inputs from, working memory has been the focus of numerous investigations to date. When considering this body of work, it is important to keep in mind the conceptual definition of Cognitive Reserve, recently clarified in the framework of the NIH-funded “Reserve and Resilience” collaboratory.^2^ The framework postulates that CR is a property or mechanism that explains cognitive performance beyond brain developmental changes and pathology, and literally reads “CR is a property of the brain that allows for cognitive performance that is better than expected given the degree of life course-related brain changes and brain injury or disease.” Cognitive Reserve is thus a *relational* construct, whose ascertainment is impossible without a cognitive endpoint and a measure that captures at least one aspect of brain health or pathology. Colloquially speaking, CR encompasses mechanisms that explain cognitive performance *beyond the influence of brain status*. In the face of this updated definition, most of the Cognitive Reserve studies to date are wanting and present a significant knowledge gap in the extant literature.

There have been numerous studies linking Cognitive Reserve to working memory. In keeping with the definition introduced before, when no brain imaging is present to quantify brain health, such studies necessarily only deal with CR *proxies*, like, for instance, education, leisure activities, occupational attainment, reading ability etc. Such factors, discussed in a recent systematic review (Song et al., 2022), *might* indeed constitute Cognitive Reserve in the sense of the framework’s stricter definition introduced above, but they cannot isolate the mediating mechanism of Cognitive Reserve conclusively beyond brain structural confounds. Superior brain health resulting in better cognition does not strictly qualify as CR and can happen in an orthogonal manner (Habeck et al., 2017). For the proper ascertainment of the presence and nature of CR, the recording of brain structural information is thus crucial but has rarely been undertaken, presenting a large gap in the field (See extensive literature review in the Discussion section.).

Since the most proximate cause of cognitive performance, and thus the obvious implementation of Cognitive Reserve, is the underlying brain activation, we wanted to repeat an age-specific derivation of load-related activation pattern, using the analytic framework in some of our earlier work in fMRI studies of verbal and non-verbal WM (Habeck et al., 2004, 2005b, 2012). We focused on the spatial activation patterns underlying the maintenance of verbal material. In contrast to our earlier work, age was considered explicitly in the analysis by looking at separate age groups, and relations between pattern scores, age, task performance, and general cognitive functioning were probed after derivation of the load-related maintenance pattern within each group.

We then wanted to test whether these activation patterns fulfilled the postulates of strict tenets of CR(Stern, 2002, 2009; Stern et al., 2019, 2020; Stern and Barulli, 2019). Since task performance was not used in the pattern derivation, the relationship of pattern utilization to task performance was likewise unconstrained. Pattern utilization could either display a positive or negative relationship with performance, or none. Significant relationships of the load-related maintenance pattern with task performance, beyond that accounted for by brain structure, would qualify as a manifestation of Cognitive Reserve. A negative sign of the relationship would indicate higher efficiency and indicate that better performing participants increase the load-related utilization of the pattern to a lesser degree than poorer performers. A positive sign, on the other hand, would indicate higher capacity in the better performers. Bivariate relationships with neuropsychological performance with the same sign as the relationship to task performance would likewise strengthen the CR interpretation.

Lastly, we were interested in widening our focus and considered the similarity of subject-level activation maps as a function of age, mean cortical thickness, task, and neuropsychological performance, without conducting any group-level pattern derivation. Beforehand, we had no expectation whether higher similarity (=lower inter-individual variability) of activation maps was associated with poorer or better performance or brain health. If conforming to a group-specific “template” is beneficial for performance, we might expect that better performing participants show higher inter-subject similarity. On the other hand, it is also conceivable, particularly for crystallized abilities, those individual neural strategies and their corresponding activation maps have been honed over a lifetime by high performers, and that consequently *lesser* inter-subject similarity might be expected. We investigated these possibilities by rigorous comparison of inter-subject similarities as a function of a variety of performance measures, age, education, and mean cortical thickness. Higher similarity can be seen as better robustness and lower variability of the topographic composition of the load-related patterns. *A priori* it was not clear whether such higher similarity would be associated positively or negatively with cognitive performance, brain structure, or younger age.

We summarize the addressable knowledge gap and its motivation for the current study: Cognitive Reserve has not been probed rigorously (i.e., comporting with the requirements of the recently funded NIH collaboratory about Reserve and Resilience) for working memory studies, and many studies have looked at the influence of *proxies*, rather than isolating mechanisms which influence cognitive performance beyond brain structural covariates. One obvious proximate mechanism that fulfills the strict Cognitive Reserve requirements would be task-related activation patterns whose pattern scores account for cognitive performance beyond brain structural variables. We aimed to test for the presence of such patterns and probe their association with traditional CR proxies, like education.

## Materials and Methods

### Participant Sample and Demographics

Analyses included data from 181 strongly right-handed, native English-speaking healthy adults. Participants were recruited *via* random market mailing and screened for MRI contraindications and hearing or visual impairment that would impede testing. Older adult participants were additionally screened to eliminate those with dementia or mild cognitive impairment. Other exclusion criteria included: myocardial Infarction, congestive heart failure or any other heart disease, and brain disorder, such as stroke, tumor, infection, epilepsy, multiple sclerosis, degenerative diseases, head injury (loss of consciousness > 5 min), intellectual disability, seizure, Parkinson’s disease, Huntington’s disease, normal pressure hydrocephalus, essential/familial tremor, Down Syndrome, HIV Infection or AIDS diagnosis, learning disability/dyslexia, ADHD or ADD, uncontrolled hypertension, uncontrolled diabetes mellitus, uncontrolled thyroid or other endocrine disease, uncorrectable vision, color blindness, uncorrectable hearing and implant, any medication targeting central nervous system, cancer within last 5 years, renal insufficiency, untreated neurosyphilis, any alcohol and drug abuse within last 12 month, recent non-skin neoplastic disease or melanoma, active hepatic disease, insulin dependent diabetes, any history of psychosis or ECT, recent (past 5 years) major depressive, bipolar, or anxiety disorder, objective cognitive impairment (dementia rating scale of <130), and subjective functional impairment (BFAS > 1). A complete description of the participants in terms of demographics and cortical thickness can be found in Table 1.

**Table 1:** Subject sample characteristics.

	Younger group	Older group	Younger ≠ Older group?
Age (mean ± STD in years)	26.0 ± 2.9	**64.8 ± 3.2**	*p*(*t*-test) < 0.0001
Education (mean ± STD in years)	15.7 ± 1.9	16.1 ± 2.4	*p*(*t*-test) = 0.11
NART-IQ (mean ± STD)	114.3 ± 7.5	**118.3 ± 8.7**	*p*(*t*-test) = 0.0058
Mean cortical thickness (mean ± STD in mm)	**2.64 ± 0.11**	2.50 ± 0.11	*p*(*t*-test) < 0.0001
Sex (#Women, #Men)	31 W, 13 M	77 W, 60 M	*p*(Fisher exact) = 0.11

Bold values indicate a statistically significant difference between age groups at *p* < 0.05.

### Neuropsychological Assessment

All participants completed a standardized battery of neuropsychological assessments, and tasks were administered in the following order: Wechsler Adult Intelligence Scale (WAIS-III; Wechsler, 1997), Letter-Number Sequencing, American National Adult Reading Test (AMNART; Wechsler, 1997), Selective Reminding Task (SRT) immediate recall (Buschke and Fuld, 1974), WAIS-III Matrix Reasoning (Wechsler, 1997), SRT delayed recall and delayed recognition (Buschke and Fuld, 1974), WAIS-III Digit Symbol (Wechsler, 1997), Trail-Making Test versions A and B (TMT-A/B; Reitan, 1978), Controlled Word Association (C-F-L) and Category Fluency (animals; Benton et al., 1983), Stroop Color Word Test (Golden, 1975), Wechsler Test of Adult Reading (WTAR; Holdnack, 2001), WAIS-III Vocabulary (Wechsler, 1997), and WAIS-III Block Design (Wechsler, 1997). Based on a prior analysis in our lab assessing the factor structure of these tasks, four domain scores were generated by *z*-scoring all tests relative to the full baseline sample, and averaging task *z*-scores within each domain: Episodic Memory (all SRT outcomes), Vocabulary (WAIS Vocabulary, WTAR, and AMNART), Processing Speed (WAIS Digit Symbol, Stroop Color, Stroop Color Word, and TMT-A), and Fluid Reasoning (WAIS Matrix Reasoning, WAIS Block Design, and TMT-B). The primary outcome measures used in the present study included the domain *z*-scores for all four cognitive domains. A further average of these four scores was performed to yield a score for total cognition (=G).

### MRI Data Acquisition

A high-resolution structural and fMRI BOLD images of the human brain were acquired in an event-related design using a 3.0 T Philips Achieva Magnet with standard quadrature head coil.

#### Structural MRI Acquisition and Processing

Each participant’s structural T1 scans were reconstructed using FreeSurfer v5.1.^3^ The accuracy of FreeSurfer’s subcortical segmentation and cortical parcellation (Fischl et al., 2002, 2004) has been reported to be comparable to manual labeling. Each participant’s white and gray matter boundaries, as well as gray matter and cerebral spinal fluid boundaries, were visually inspected slice by slice, and manual control points were added in case of any visible discrepancy. Boundary reconstruction was repeated until satisfactory results for every participant were reached. The subcortical structure borders were plotted by TkMedit visualization tools and compared against the actual brain regions. In case of discrepancy, they were corrected manually. We took the mean cortical thickness measures reported for both hemispheres and averaged them, to arrive at one global measure per participant.

#### fMRI Acquisition of the Sternberg Working Memory Task

Functional data were acquired in three runs, each of which included collection of 314 functional volumes using a T2*-weighted gradient-echo echo planar image sequence. About 36 transverse slices per volume with 3.0 mm thickness and no gap in between were acquired using a field echo echo planar imaging (FE–EPI) sequence with the following parameters: TR 2,000 ms, TE 20 ms, and flip angle 72; in-plane acquisition matrix 112 × 112 matrix; which results in a voxel size 2.0 mm × 2.0 mm × 3.0 mm.

Task stimuli were back-projected onto a screen located at the foot of the MRI bed using an LCD projector. Participants viewed the screen *via* a mirror system located in the head coil and, if needed, had vision corrected to normal using MR compatible glasses (manufactured by SafeVision, LLC. Webster Groves, MO, United States). Task administration and collection of behavioral data were conducted using PsyScope 5X B53 (Macwhinney et al., 1997). Task onset was electronically synchronized with the MRI acquisition computer.

### Letter Sternberg task

Participants studied an array of either one, three, or six upper-case letters for 3 s (=encoding phase), then a fixation screen was presented for 7 s (=maintenance phase), to be probed with a lower-case letter, presented for 3 s. Participants were told to respond as quickly as possible with a differential button press whether the letter was part of the studied array or not (=retrieval phase). The probe letter remained on screen for the full 3 s regardless of response time. For the current study, we only focused on the maintenance phase.

### Sternberg Task fMRI Data Processing

#### Subject-Level Pre-processing

FMRIB Software Library v5.0 (FSL) and custom-written Python code were used to perform the following pre-processing steps for each participant’s dataset: All functional images were realigned to the first volume, corrected for the order of slice acquisition, smoothed with a 5 mm^3^ non-linear kernel followed by intensity normalization, and high-pass filtered using a Gaussian kernel and cutoff frequency of 0.008 Hz. For spatial normalization, the accompanying T1-weighted high-resolution anatomic image was co-registered to the first functional volume using the mutual information co-registration algorithm implemented in FLIRT. This co-registered high-resolution image was then registered to MNI standardized space. These obtained transformation parameters were used to transfer the statistical parametric maps of the subject-level analysis to standard space.

The fMRI time series data were pre-whitened to explicitly correct for intrinsic autocorrelations in the data. The FEAT module (Woolrich et al., 2001) in FSL was used for first-level analysis. An event-related design was used to model the fMRI data, allowing us to separate timeouts (where no response was made), correct and incorrect trials, task loads (Braver et al., 1997; Jonides et al., 1997; Smith and Jonides, 1998), and task phases (encoding, maintenance, and retrieval). Incorrect responses and timeouts were modeled together. For all participants, a first-level analysis was run on each of their task-based runs with nine regressors: 3 task loads × 3 task phases. The regressors were generated by convolving FSL’s double gamma canonical HRF with the duration of the respective task phases: encoding = 3 s, maintenance = 7 s, and retrieval = RT. A second level analysis was run on each participant by combining the first-level contrasts for each run. Contrasts for the retention phase of the three memory loads, 1, 3, and 6, were used in subsequent analysis.

### Ordinal Trend Canonical Variates Analysis and Brain-Behavioral Analysis

We first identified a memory load-related activation pattern during the retention (=maintenance) period. We applied Ordinal Trend Canonical Variates (OrT-CVA) analysis (Habeck et al., 2004, 2005a) to derive a group-level activation pattern that shows an increase in pattern expression during the retention period with memory load on an individual subject level. We can write the multivariate decomposition achieved by OrT-CVA as follows. The derived activation pattern will be written as *v*, and participant S’s activation map for memory load L can be written as the indexed column vector *y*(S,L). This activation map can be written as the product of the group-level activation pattern with a subject- and load-dependent factor score w(S,L) and some unaccounted residual ε:


$$y\left(S,L\right)=w\left(S,L\right)\;v+\varepsilon$$

Ordinal Trend Canonical Variates puts constraints on the factor score *w*(S,L) and derives an activation pattern whose factor score shows a positive within-person relationship with memory load for as many participants as possible, with an inferential framework for ascertaining significance through a permutation test.

For the estimation of topographic robustness, a bootstrap estimation procedure was conducted which resampled all participants and performed the OrT-CVA point estimate procedure on the resampled data 500 times. A topographic *Z*-map was approximated semi-parametrically by computing the bootstrap variability as a SD around the point estimate, and dividing the point estimate by this SD as.


$$Z\left(\mathrm{voxel}\right)=\mathrm{point estimate loading}\;\left(\mathrm{voxel}\right)/STD\;\left(\mathrm{voxel}\right)$$

A minimum value of |*Z*| > 2 is required for regions to be highlighted with a consistent loading in a visualization plot. We performed a bootstrap procedure with 500 iterations and resampled the data with replacement, repeating the complete OrT-CVA analytic stream to compute *Z*-maps quantifying the robustness of each voxel’s contribution to the covariance pattern. In addition to the group-specific robustness *Z*-maps, we also computed all possible 500 × 500 = 250,000 difference maps and looked at the voxels which lay in the 95% tail of the difference distribution, flagging significant differences in the loadings between young and old participants.

### Relationship Between Regional Cortical Thickness and Sternberg Task Performance

We performed region-wise univariate analysis and related cortical thickness to Sternberg task performance in both age groups separately and identified a set of regions whose thickness shows a negative association with load-averaged reaction time, and a set of regions whose thickness shows a positive association with load-averaged accuracy. We used a liberal threshold of *p* < 0.05, and then combined all identified regions with this liberal screen into one linear combination through linear regression analysis. In the end, we thus ended up with four thickness-based estimates of task performance for two outcomes and in two age groups. These thickness-based performance estimates where used in any subsequent brain-behavioral analysis involving the derived load-related activation pattern, to maximize the variance accounted for in the task performance variables by cortical thickness.

### Inter-Pattern Similarity in the Older Group as a Function of Performance, Education, and Mean Cortical Thickness

In the next section, we tried to address the question whether age or education causes smaller or larger inter-individual variability, and whether or how good cognitive performance or preserved brain structure can offset any differences of activation patterns between individuals. For age, education, and cortical thickness, we just performed a median split of the older participants. For neuropsychological and task performance, to adjust for confounding factors, we residualized these measures regarding age, education, sex, and cortical thickness performance estimate, and then split the residuals along the respective median. Both procedures resulted in an assignment of each older participant into a “high” and “low” group with respect to each variable of interest. Our question was whether inter-individual similarity in activation maps differed between the “high” and “low” group, that is, we wanted to test the null-hypothesis:


$$R_{\mathrm{high}}\left(k,j\right)=R_{\mathrm{low}}\left(m,n\right)$$

For all subject pairs, (*k*,*j*) in “high” and (*m*,*n*) in “low”

For this test, we computed all possible N*(*N*-1)/2 pairwise inter-subject spatial correlation coefficients (where *N* is 68 and 69, respectively). These inter-spatial correlation coefficients can be contrasted with a simple *t*-test for a point estimate computation; however, to assess statistical significance, a permutation test is needed that repeats the inter-subject similarity computation for null conditions. That is, we permuted participants randomly between “high” and “low” groups, creating null conditions, and repeating the within-group inter-subject similarity computation and the computation of the group comparison T-statistic. This was done 1,000 times to create a null-histogram, and statistical significance was approximated with a two-tailed test as *P*(|*T*(permutation)| > |*T*(point estimate)|). This procedure is necessary since a parametric test would overestimate the number of the degrees of freedom and the *T*-statistic itself, and consequently suffer from value of *p* inflation with nominal value of *p* that would be too small.

## Results

**Figure 1:**
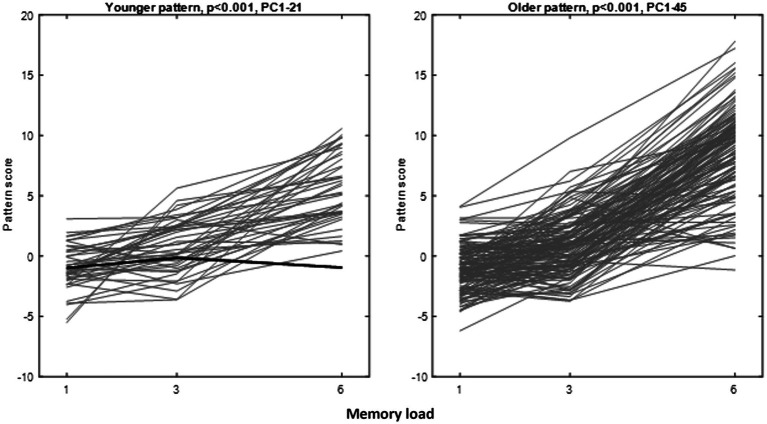
Pattern score curves for the Ordinal Trend (OrT) patterns derived for younger and older participants. Exceptions to the positive-slope rule are shown in bold face. There was one exception in the young group, but none in the older group.

We identified reliable load-related patterns during the retention phase in both age groups. In the younger participants, a best-fitting load-related pattern was constructed from PCs 1–21 and one of the participants did not conform to the majority rule of positive expression slopes (*p* < 0.001, permutation test). In the older participants, we constructed a pattern from PCs 1–45, and nobody deviated from the majority positive-slope rule (*p* < 0.001, permutation test). Figure 1 shows the task–activity curve for every participant in both age groups.

The OrT-CVA technique imposes no *a priori* relationship between load-related slopes and load averages of pattern scores, and any relationship (negative, positive, or no correlation) is conceivable *a priori*, which we verified the correctness of parametric assumptions with permutation tests. For both groups, the load-related pattern score slope was positively correlated with the mean pattern score (Young: *R* = 0.4143, *p* = 0.0052; Old: *R* = 0.5048, *p* < 0.0001). Participants manifesting higher pattern score slopes thus also showed higher overall pattern scores. This finding refuted possible ceiling effects, with task–activity curves starting at relatively high levels with consequently lesser load increases.

We related the load average and slope of the pattern expression scores to Sternberg task performance (load average and slope of accuracy and reaction time) and four domains of neuropsychological functioning in bivariate correlations and found notably different brain-behavioral correlations as a function of age. We only report significant correlations: in the younger group, the pattern expression slope correlated negatively with mean load-averaged task accuracy in the Sternberg task (*R* = −0.32531, *p* = 0.031183), fluid reasoning (*R* = −0.37756, *p* = 0.011516), and vocabulary (*R* = −0.39524, *p* = 0.0079228). In the older group, pattern slope correlated positively with mean Sternberg task accuracy (*R* = 0.17672, *p* = 0.038851), and the mean pattern score correlated positively with mean Sternberg task accuracy (*R* = 0.28577, *p* = 0.00071152), perceptual speed (*R* = 0.21227, *p* = 0.012767), and vocabulary (*R* = 0.21027, *p* = 0.013655).

**Figure 2:**
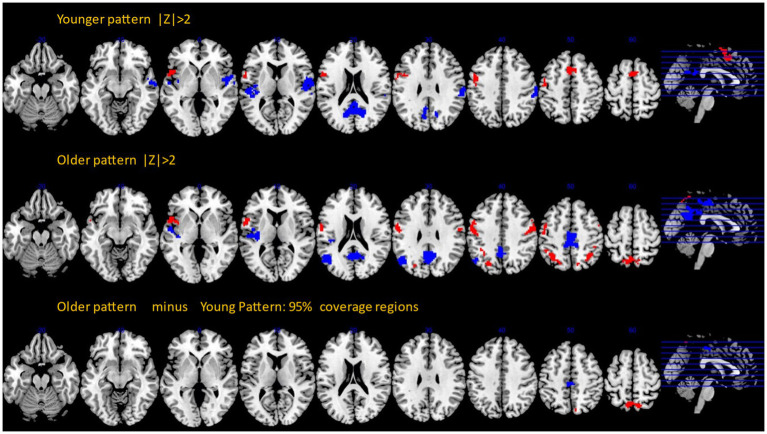
Bootstrap *Z*-maps (top and middle rows) thresholded at |*Z*| > 2, cluster size > 50. Bottom row: inferential comparison of old and young patterns, showing areas with the 95% coverage intervals excluding zero values.

We performed a bootstrap procedure and computed *Z*-maps quantifying the robustness of each voxel’s contribution to the covariance pattern, as well as inferential difference maps identifying old–young differences with 95% confidence. The results are shown in Figure 2. Topographic composition of both patterns shared a lot of similarities but involved two stark differences showing additional involvement of areas in the older participants: the involvement of the posterior cingulate for negative loadings and bilateral precuneus for positive loadings (Supplementary Table 3).

Super-threshold regions for all three images with anatomical annotation are listed in Supplementary Tables 1–3.

We also related regional cortical thickness to load-averaged reaction time and accuracy in both age groups, see Table 2.

**Table 2:** Relationships between regional cortical thickness and task performance on Sternberg task.

Endpoint and age group	FreeSurfer label of associated regions at *p* < 0.05
Reaction time in younger group (negative association)	lh-isthmuscingulate, rh-medialorbitofrontal
Reaction time in older group (negative association)	lh-inferiortemporal, lh-medialorbitofrontal, lh-middletemporal, lh-parahippocampal, lh-precuneus, lh-temporalpole, lh-insula, rh-entorhinal, rh-fusiform, rh-inferiortemporal, rh-lateraloccipital, rh-lateralorbitofrontal, rh-middletemporal, rh-parahippocampal, rh-temporalpole, and rh-insula
Accuracy in younger group (positive association)	lh-paracentral, lh-postcentral, lh-precentral, lh-supramarginal, lh-transversetemporal, rh-inferiorparietal, rh-postcentral, and rh-transversetemporal
Accuracy in older group (positive association)	lh-isthmuscingulate, lh-posteriorcingulate, and rh-isthmuscingulate

Apart from bivariate correlations, we also ran linear regressions to predict load-averaged Sternberg accuracy rates with pattern scores, demographics, and the performance estimate based on cortical thickness as covariates. Table 3 below revealed the importance of general intelligence as measured by G since it contributed to task performance above all other measures. Only in the younger group did the mean pattern score of the load-related pattern also contribute to task performance; in the older group, significance of this association was only marginally significant with *p* = 0.0665 (We performed corresponding linear regressions to predict mean reaction time in both age groups but did not find any associations with the load-related patterns in either age group and elected to omit the table listing for clarity.).

**Table 3:** Mean load-averaged task accuracy as a function of pattern-score mean levels and slopes, neuropsychological functioning, and demographics.

Outcome: mean task accuracy	Young		Old	
	*T*-statistic	*p* value	*T*-statistic	*p* value
Intercept	0.7531	0.4563	0.4838	0.6293
Mean pattern score	**2.2210**	**0.0327**	1.8505	0.0665
Pattern score slope	−1.4905	0.1448	1.0507	0.2954
Thickness-based accuracy estimate	**3.1379**	**0.0034**	**2.3689**	**0.0193**
Age	1.6710	0.1034	0.8319	0.4070
Total G	**2.4477**	**0.0194**	**3.5071**	**0.0061**
Education	−1.0555	0.2982	−0.3157	0.7527
Sex	0.5569	0.5810	−0.1460	0.8842

Pattern scores can account for task accuracy beyond the covariates with only marginal *p* value in the older participants. Total cognition (=G) account for performance beyond cortical thickness too.

Bold values indicate a statistically significant difference between age groups at *p* < 0.05.

We decided to run an *ad hoc* analysis and project the pattern derived in the younger group into the activation data of the older group to examine the degree that older adults express the younger adults’ activation pattern. Multivariate patterns possess this convenient feature and enable simple cross-applications to *de novo* data. When computing bivariate correlations between subject variables with the obtained pattern scores like before, we found positive associations between the mean load-averaged pattern score and task performance (*R* = 0.20946, *p* = 0.014029) as well as perceptual speed (*R* = 0.20802, *p* = 0.014719).

**Figure 3:**
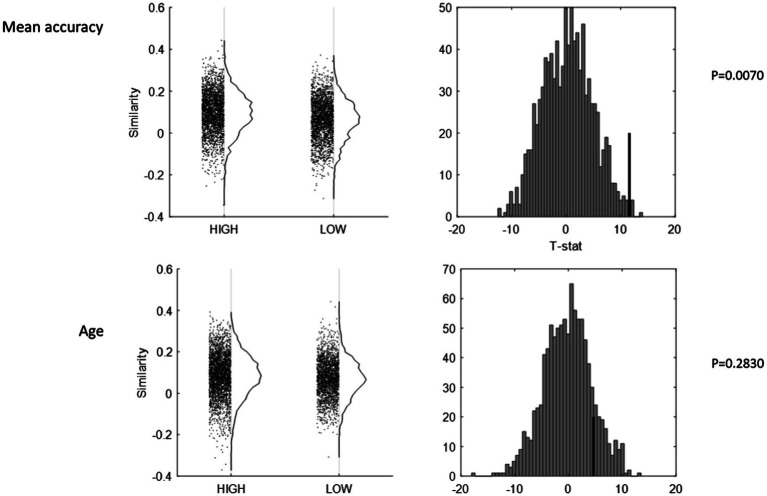
Inter-subject pattern similarity as a function of mean task accuracy (top row), and age (bottom row). All inter-subject similarity values are plotted against HIGH and LOW status (left column), with null-histograms for the group contrast from a permutation test of 1,000 iterations (right column). The group with higher task accuracy showed higher inter-subject similarity (*p* = 0.0070), whereas age was non-differential (*p* = 0.2830).

Next, we turned to the dichotomization of the older group along several median splits based on task performance, neuropsychological functioning, education, and mean cortical thickness and observed the group differences in the inter-subject similarity of mean activation patterns. We found no significant differences between “high” and “low” groups for age, education (shown in Figure 3), mean cortical thickness, or task reaction time (minimum value of *p* for four comparisons from permutation test: 0.227). Significantly higher similarity was found for the “high” group for mean task accuracy (*T* = 11.6054, *p* = 0.0070, shown in Figure 3), memory (*T* = 11.8313, *p* = 0.0060), fluid reasoning (*T* = 12.9849, *p* = 0.0008), speed (*T* = 9.7044, *p* = 0.0300), and vocabulary (*T* = 11.9092, *p* = 0.0090).

Although it was only of secondary interest, we also performed several comparisons of inter-subject similarity involving the young participants, comparing all young and old participants. We found significantly higher inter-subject similarity for old compared to young adults (*T* = 10.1118, *p* = 0.034). When we revisited the median splits in the older participants, this difference was only found for the contrast of young adults vs. high-performing older adults. This hinted at inverted u-shaped behavior of inter-subject similarity regarding age-related performance: inter-subject similarity was low for young and low-performing older participants on the two ends, but high for high-performing older participants in the middle.

## Discussion

We identified load-related activation patterns during the maintenance phase of a verbal working memory task in two age groups that showed relationships to neuropsychological functioning and task performance, even though these relationships differed starkly as a function of age. In the young-participant group, the load-related increases, that is, the slope of pattern scores, related *negatively* to neuropsychological functioning and mean task accuracy, that is, poor performers increased their pattern score in response to memory load increases to a greater degree. Apparently, the younger participant’s rehearsal pattern can be interpreted as *necessary*, but greater engagement of it in response to memory load was *not* conducive to good performance. In the older participant group, on the other hand, the load-related slope of pattern scores related positively to mean accuracy, as did the mean load-averaged pattern scores, which also correlated positively with neuropsychological functioning. Further, when prospectively applying the young-derived activation pattern into the older group’s activation maps, the resulting mean load-averaged pattern scores still displayed positive correlations with mean task accuracy neuropsychological functioning (=perceptual speed). If the young-derived activation pattern can be considered as a “template,” then higher manifestation of this template in the older groups is associated with better task performance and better perceptual speed. The young activation pattern can be considered an implementation of Cognitive Reserve since load-averaged pattern scores accounted for task performance beyond cortical thickness, general cognition, and demographics; the activation pattern from the older group only achieved marginal significance in this regard. One caveat is that cortical thickness is only *one* possible measure of brain health and ideally a more holistic assessment which integrates several modalities, like gray matter volume and thickness, white-matter integrity, and absence of amyloid and tau protein accumulation, should be used.

Our study started to address a significant knowledge gap in the field. While there have been numerous studies invoking the concept of Cognitive Reserve, few of them comported with the strict definition of the recent clarification in the NIH-funded initiative “Reserve and Resilience.”^4^ Behavioral studies have demonstrated the link between working memory and general cognition in many different forms. Questionnaire-based operationalization of CR has shown correlations with working memory performance in patients with subject memory complaints (Lojo-Seoane et al., 2020), education has been shown to predict performance on an N-back task (Zarantonello et al., 2020), while vocabulary ability reduced working memory differences between participants with single- and multi-domain Mild Cognitive Impairment (Facal et al., 2014). In a community sample, verbal intelligence had stronger associations with working memory than education or socioeconomic status (Jefferson et al., 2011). For cognitive interventions, such as training on the N-back task, training-related improvements (Mičič et al., 2020) correlated with a measure operationalized through the Cognitive-Reserve Index questionnaire (Nucci et al., 2012). Working memory itself could be a form of Cognitive Reserve, as shown in a study (Sandry and Sumowski, 2014) that looked at intellectual enrichment and long-term memory outcomes in multiple sclerosis patients and found independent contributions by working memory capacity and, crucially, a moderation of the relationship between intellectual enrichment and long-term memory outcomes.

A different class of studies has related CR proxies to functional brain signals, without simultaneous consideration of brain structure or pathology. These studies often establish robust functional correlates of CR proxies, while still following short of the rigor required by the “Reserve and Resilience” framework. Event-related potentials (ERPs) recorded with electro-encephalography can link latency and amplitude of the signal to working memory performance or CR proxies with suggestive findings for increased efficiency with, for instance, reading ability (Gutiérrez-Zamora Velasco et al., 2021) or verbal intelligence and education (Speer and Soldan, 2015). While these studies clearly demonstrate ERP correlates of CR proxies, a rigorous ascertainment of CR cannot be established because of the absence of brain structure or pathology measures. Even when such information is present, the full analyses required to decide about CR mechanisms are not always run. For instance, an fMRI study of a two-back task (Bartrés-Faz et al., 2009) showed correlations between fMRI activation and a composite of education, occupation, verbal ability, and leisure activities, supporting the notion of increased processing efficiency, but did not directly use the structural covariate of regional gray matter volume in a combined analysis. Similarly, in a recent fMRI lifespan study (Archer et al., 2018) of a spatial WM task where regional gray matter volume was used as a structural covariate, rigorous tests of Cognitive Reserve conforming to the framework’s standards were lacking. The study showed negative associations between task-related activation and age in a monotonic fashion and thus substantiated age-related decreases in processing efficiency, although adjustments for education rendered some of these relationships non-significant. Crucially, the test of any contribution of education to task performance beyond the study’s covariate of choice, *gray matter volume*, necessary to verify education’s role as a Cognitive Reserve mechanisms, was also not conducted. Education was shown to be associated positively with task performance in bivariate correlations, but without the clarification of the role of gray matter volume, a clear differentiation between brain maintenance versus Cognitive Reserve effects, again, could not be made in the characterization of the education effect.

Other interesting studies that capture the spirit, if not the full operationalization, of the “Reserve and Resilience” framework can be found when the effect of general health impediments is not explicitly localized in the brain but can be shown to have a detrimental effect on working memory, with a moderation of the health-cognition relationship by CR proxies. In one study (Ihle et al., 2018), hypertension lowered working memory performance, but this effect was lessened when education, occupational demands, and leisure activities were considered too.

In conclusion, we can say studies that have investigated working memory and possible CR mechanisms with an adequate account brain structure or pathology are thus quite sparse in the extant literature. A recent study of the effect of bilingualism on performance of a two-back task (Anderson et al., 2021) showed structure–cognition associations that differed between bilinguals and monolinguals, which constitute a rigorous test of Cognitive Reserve consistent with the NIH-funded “Reserve and Resilience” framework. Similarly, after matching dyads of participants on hippocampal volume, one study showed that education was positively associated with working memory performance, among several cognitive outcomes (Rodriguez et al., 2019). Lastly, verbal intelligence has been shown to lessen the detrimental effect of amyloid deposition on working memory (Rentz et al., 2010).

After deriving task-related activation patterns and confirming their fulfillment of the Cognitive Reserve postulates, we also investigated the inter-subject similarity of load-averaged activation patterns underlying the maintenance phase as a function of age, task and neuropsychological performance, education, and mean cortical thickness. Younger adults had lower inter-subject similarity than older adults, hinting at a narrowing of individual neural strategies with age. In the older group however, *higher* inter-subject similarity was associated with *better* performance (but *not* longer education). Both young and low-performing older adults presented with lower inter-subject similarity than high-performing older adults.

This non-monotonic behavior of inter-subject similarity (=topographic robustness) was somewhat surprising. Inter-individual variability has not been researched extensively, and intra-individual variability has received the most of the field’s recent attention (Armbruster-Genc et al., 2016; Nomi et al., 2017; Grady and Garrett, 2018; Pur et al., 2019; Roberts et al., 2020). Within a person and region, these studies broadly suggest that increased task demand and better cognitive performance might cause increased fMRI variability, and age might negatively impact cognitive performance *via* both depressed variability overall and the lower ability to increase variability in response to task demands. The implications of this research for inter-individual variability are not clear: inter-individual variability indicates that different people are employing different neural substrates, leading to lower inter-subject similarity. This reduced inter-subject variability can be seen as a group-level manifestation of a Cognitive Reserve mechanism: high performers seem to involve more similar individual-level activation maps than poorer performs. Since our findings are cross-sectional they can suggest plausibility, while longitudinal lifespan studies can more rigorously disentangle aging from age effects for any neural substrates, including inter-subject variability.

In summary, our study illustrated two facets of Cognitive Reserve and resilience in aging. We identified a memory load-related maintenance pattern that was positively associated with performance and neuropsychological functioning, but not with cortical thickness, in older participants. Interestingly, in younger participants, a topographically similar pattern could be identified, but the performance relations were strikingly different: higher deployment of the pattern in response to memory load was associated with *worse* performance on the task and *worse* neuropsychological functioning, hinting at a possible critical age range at which this change might occur, to be investigated more thoroughly in lifespan data with continuous age coverage. The different relations to performance notwithstanding, the pattern derived from young participants fulfilled the tenets of Cognitive Reserve and its mean load-averaged pattern scores were associated with task performance beyond brain structure, while the pattern derived from older participants achieved marginal significance. High inter-subject similarity of activation maps, which was assessed outside a group-level analytic framework, can be considered a form of (group-level) Cognitive Reserve as well, at least in the older participants, and is associated with more robust group-level activation patterns too. This suggests that better cognitive functioning might converge on *one* optimal neural strategy, deviations from which might be suboptimal in older age. An alternative scenario might be one of *neural flexibility* at the group-level, that is, a scenario where high-performing participants have honed their individual neural strategies and use *different* activation patterns from each other, hampering an effective group-level analysis strategy. Covariance analysis in general can accommodate differences *in degree* along a group-invariant construct (=activation pattern) but cannot accommodate differences *in kind* very effectively. If high performers showed greater inter-subject variation in the topographic composition of their individual-level activation maps, tailoring a group-level pattern would involve an increased numbers of principal components, with correspondingly lower inferential robustness.

Such group-level variability with *positive* association to Cognitive Reserve might be conceivable for other cognitive tasks beyond working memory; investigation of inter-subject similarity and group-level robustness and its association with Cognitive Reserve, age and aging in longitudinal lifespan data will remain on our agenda for the near future.

## Data Availability Statement

The datasets presented in this study can be found in online repositories. The names of the repository/repositories and accession number(s) can be found at: Dryad Repository, final DOI: 10.5061/dryad.bzkh189c3. Preliminary access: https://datadryad.org/stash/share/gQSaYyiwz9he9_KI37ufnZpSLZU8VrzKAa8wMnYIdJU.

## Ethics Statement

The studies involving human participants were reviewed and approved by Human Research Protection Office and IRB, Columbia University. The patients/participants provided their written informed consent to participate in this study.

## Author Contributions

CH: conceptualization, analysis, and manuscript writing. YG: data pre-processing and manuscript writing and editing. YS: manuscript writing and editing and study design. All authors contributed to the article and approved the submitted version.

## Funding

Funding is gratefully acknowledged from grant: NIH R01AG026158.

## Conflict of Interest

The authors declare that the research was conducted in the absence of any commercial or financial relationships that could be construed as a potential conflict of interest.

## Publisher’s Note

All claims expressed in this article are solely those of the authors and do not necessarily represent those of their affiliated organizations, or those of the publisher, the editors and the reviewers. Any product that may be evaluated in this article, or claim that may be made by its manufacturer, is not guaranteed or endorsed by the publisher.

## Footnotes

^2^ https://reserveandresilience.com/framework/

^3^ http://surfer.nmr.mgh.harvard.edu/

^4^ https://reserveandresilience.com/
