# Supplementary material for: Age-Specific Activation Patterns and Inter-Subject Similarity During Verbal Working Memory Maintenance and Cognitive Reserve
Source: Front Psychol. 2022 Jun 9;13:852995. doi: 10.3389/fpsyg.2022.852995 (PMC9218333; doi:10.3389/fpsyg.2022.852995)
Supplement: Supplementary file 1 [file Table_1.DOCX]

**Supplementary Table 1:** Robust areas in Z-map of younger participants, |Z|>2, cluster size >100.

| MNI-X | MNI-Y | MNI-Z | \|Cluster\| | Z-value | AAL_label |
| --- | --- | --- | --- | --- | --- |
| Positive loadings | | | | | |
| -51 | -3 | 48 | 486 | 3.6577 | Precentral_L |
| -3 | 15 | 51 | 292 | 3.587 | Supp_Motor_Area_L |
| -48 | -12 | 51 | 486 | 3.572 | Postcentral_L |
| -3 | 6 | 60 | 292 | 3.5712 | Supp_Motor_Area_L |
| -57 | 6 | 12 | 486 | 3.477 | Rolandic_Oper_L |
| -36 | 45 | 30 | 129 | 3.2676 | Frontal_Mid_L |
| -39 | 21 | 27 | 129 | 3.2521 | Frontal_Inf_Tri_L |
| -45 | 6 | 30 | 486 | 3.2491 | Frontal_Inf_Oper_L |
| -54 | 9 | 27 | 486 | 3.2278 | Precentral_L |
| -54 | 9 | 3 | 486 | 3.227 | Rolandic_Oper_L |
| -39 | -3 | 60 | 486 | 3.1612 | Precentral_L |
| -30 | 15 | 9 | 486 | 3.0126 | Insula_L |
| -39 | 18 | 0 | 486 | 3.0108 | Insula_L |
| -6 | 21 | 36 | 292 | 2.936 | Cingulum_Mid_L |
| -39 | 33 | 39 | 129 | 2.926 | Frontal_Mid_L |
| -39 | 33 | 21 | 129 | 2.8969 | Frontal_Inf_Tri_L |
| -60 | 0 | 30 | 486 | 2.8578 | Precentral_L |
| 9 | 27 | 42 | 292 | 2.7099 | Cingulum_Mid_R |
| -30 | 54 | 18 | 129 | 2.7044 | Frontal_Mid_L |
| -3 | 30 | 39 | 292 | 2.6549 | Frontal_Sup_Medial_L |
| -33 | 39 | 24 | 129 | 2.5351 | Frontal_Mid_L |
| -42 | 6 | 3 | 486 | 2.4289 | Insula_L |
| 15 | 12 | 66 | 292 | 2.1736 | Supp_Motor_Area_R |
| -24 | -3 | 66 | 486 | 2.169 | Frontal_Sup_L |
| 6 | -9 | 66 | 292 | 2.1444 | Supp_Motor_Area_R |
| -51 | 24 | 30 | 129 | 2.0759 | Frontal_Inf_Tri_L |
| Negative loadings | | | | | |
| -15 | -66 | 21 | 529 | -3.4581 | Cuneus_L |
| 21 | -60 | 24 | 529 | -3.4547 | Precuneus_R |
| 12 | -57 | 18 | 529 | -3.4359 | Calcarine_R |
| -6 | -54 | 21 | 529 | -3.4349 | Precuneus_L |
| 51 | 0 | 0 | 534 | -3.3336 | Temporal_Sup_R |
| 9 | -63 | 24 | 529 | -3.3258 | Precuneus_R |
| 66 | -9 | 6 | 534 | -3.3014 | Temporal_Sup_R |
| 51 | -21 | 12 | 534 | -3.2 | Temporal_Sup_R |
| 57 | -24 | 30 | 534 | -3.1932 | SupraMarginal_R |
| 0 | -45 | 42 | 529 | -3.1197 | Precuneus_R |
| 57 | -9 | -12 | 534 | -3.0828 | Temporal_Mid_R |
| 51 | -48 | 27 | 152 | -3.0715 | Angular_R |
| 36 | -27 | 15 | 534 | -3.041 | Heschl_R |
| -39 | -9 | 6 | 235 | -2.9934 | Insula_L |
| -54 | -6 | 0 | 235 | -2.9891 | Temporal_Sup_L |
| 3 | -45 | 21 | 529 | -2.9823 | Precuneus_R |
| 60 | -51 | 3 | 152 | -2.9715 | Temporal_Mid_R |
| 42 | -3 | 3 | 534 | -2.9607 | Insula_R |
| -36 | -24 | 9 | 235 | -2.9233 | Heschl_L |
| -54 | -24 | 9 | 235 | -2.9023 | Temporal_Sup_L |
| 42 | 0 | -12 | 534 | -2.857 | Insula_R |
| 45 | -66 | 9 | 152 | -2.7692 | Temporal_Mid_R |
| 51 | -57 | 24 | 152 | -2.6857 | Temporal_Sup_R |
| 63 | -12 | -6 | 534 | -2.6635 | Temporal_Sup_R |
| -24 | 6 | -24 | 235 | -2.6585 | ParaHippocampal_L |
| 45 | -63 | 18 | 152 | -2.6308 | Temporal_Mid_R |
| 45 | -63 | 36 | 152 | -2.596 | Angular_R |
| -3 | -54 | 42 | 529 | -2.5906 | Precuneus_L |
| 66 | -27 | 9 | 534 | -2.5749 | Temporal_Sup_R |
| 12 | -54 | 39 | 529 | -2.5624 | Precuneus_R |
| -12 | -39 | 45 | 529 | -2.4632 | Precuneus_L |
| 63 | -15 | 39 | 534 | -2.4596 | Postcentral_R |
| 12 | -45 | 51 | 529 | -2.4494 | Precuneus_R |
| -6 | -36 | 33 | 529 | -2.359 | Cingulum_Post_L |
| 48 | 9 | -15 | 534 | -2.3583 | Temporal_Pole_Sup_R |
| 60 | -42 | 12 | 152 | -2.2212 | Temporal_Mid_R |
