# Supplementary material for: Age-Specific Activation Patterns and Inter-Subject Similarity During Verbal Working Memory Maintenance and Cognitive Reserve
Source: Front Psychol. 2022 Jun 9;13:852995. doi: 10.3389/fpsyg.2022.852995 (PMC9218333; doi:10.3389/fpsyg.2022.852995)
Supplement: Supplementary file 2 [file Table_2.DOCX]

**Supplementary table 2:** Robust areas in Z-map of older participants, |Z|>2, cluster size >100

| MNI-X | MNI-Y | MNI-Z | \|Cluster\| | Z | AAL_label |
| --- | --- | --- | --- | --- | --- |
| Positive loadings | | | | | |
| -51 | -12 | 48 | 518 | 3.5701 | Postcentral_L |
| 57 | -6 | 45 | 100 | 3.5403 | Precentral_R |
| -3 | 3 | 57 | 116 | 3.4214 | Supp_Motor_Area_L |
| 36 | -45 | 42 | 225 | 3.398 | Parietal_Inf_R |
| -48 | 0 | 51 | 518 | 3.3415 | Precentral_L |
| -24 | -66 | 39 | 409 | 3.2795 | Occipital_Mid_L |
| 48 | -9 | 39 | 100 | 3.2772 | Postcentral_R |
| 33 | -54 | 48 | 225 | 3.2121 | Angular_R |
| -51 | 12 | -3 | 518 | 3.1655 | Temporal_Pole_Sup_L |
| -36 | -51 | 48 | 409 | 3.155 | Parietal_Inf_L |
| 30 | -66 | 36 | 225 | 3.1418 | Occipital_Mid_R |
| -60 | -3 | 21 | 518 | 3.1369 | Postcentral_L |
| -33 | -45 | 42 | 409 | 3.1224 | Parietal_Inf_L |
| -21 | -66 | 48 | 409 | 3.0988 | Parietal_Sup_L |
| -51 | 6 | 39 | 518 | 3.0645 | Precentral_L |
| -27 | -72 | 30 | 409 | 3.0442 | Occipital_Mid_L |
| 3 | -81 | 9 | 185 | 3.0263 | Calcarine_R |
| -33 | -63 | 54 | 409 | 3.0015 | Parietal_Sup_L |
| -33 | 12 | 9 | 518 | 2.9373 | Insula_L |
| -21 | -60 | 63 | 409 | 2.9311 | Parietal_Sup_L |
| -9 | -81 | 9 | 185 | 2.9071 | Calcarine_L |
| -6 | -66 | 60 | 409 | 2.8916 | Precuneus_L |
| 54 | 3 | 45 | 100 | 2.8479 | Precentral_R |
| 30 | -66 | 54 | 225 | 2.8314 | Parietal_Sup_R |
| -45 | -36 | 45 | 409 | 2.7427 | Parietal_Inf_L |
| -3 | -57 | 63 | 409 | 2.7303 | Precuneus_L |
| 15 | -75 | 9 | 185 | 2.7062 | Calcarine_R |
| -9 | -72 | 9 | 185 | 2.7035 | Calcarine_L |
| -51 | 12 | 30 | 518 | 2.6753 | Frontal_Inf_Oper_L |
| -30 | 27 | 0 | 518 | 2.6596 | Insula_L |
| -60 | 3 | 9 | 518 | 2.6387 | Rolandic_Oper_L |
| -36 | 6 | 3 | 518 | 2.6374 | Insula_L |
| -45 | 21 | 21 | 518 | 2.6332 | Frontal_Inf_Tri_L |
| 3 | -81 | 27 | 185 | 2.614 | Cuneus_L |
| 3 | -69 | 57 | 409 | 2.6057 | Precuneus_R |
| -48 | 9 | 15 | 518 | 2.5764 | Frontal_Inf_Oper_L |
| 36 | -72 | 27 | 225 | 2.5223 | Occipital_Mid_R |
| 12 | -87 | 3 | 185 | 2.4569 | Calcarine_R |
| 3 | 24 | 45 | 116 | 2.4526 | Frontal_Sup_Medial_R |
| -15 | -51 | 63 | 409 | 2.4507 | Precuneus_L |
| 9 | -75 | 51 | 409 | 2.45 | Precuneus_R |
| 15 | -69 | 54 | 409 | 2.4096 | Parietal_Sup_R |
| 57 | 9 | 36 | 100 | 2.3679 | Precentral_R |
| -3 | -93 | 3 | 185 | 2.3531 | Calcarine_L |
| 6 | -63 | 63 | 409 | 2.3393 | Precuneus_R |
| 51 | -33 | 45 | 225 | 2.3064 | SupraMarginal_R |
| 51 | -33 | 54 | 225 | 2.3059 | Parietal_Inf_R |
| -45 | 30 | 36 | 518 | 2.2922 | Frontal_Mid_L |
| -6 | -69 | 51 | 409 | 2.2407 | Precuneus_L |
| -42 | 18 | 30 | 518 | 2.1814 | Frontal_Inf_Tri_L |
| Negative loadings | | | | | |
| -6 | -54 | 30 | 484 | -3.6672 | Precuneus_L |
| -36 | -24 | 12 | 169 | -3.256 | Heschl_L |
| -48 | -72 | 36 | 255 | -3.2321 | Angular_L |
| -12 | -33 | 48 | 232 | -3.1349 | Cingulum_Mid_L |
| -45 | -63 | 18 | 255 | -3.1079 | Temporal_Mid_L |
| 54 | -6 | 6 | 292 | -3.0981 | Heschl_R |
| 9 | 0 | 45 | 232 | -3.0871 | Cingulum_Mid_R |
| -6 | -42 | 36 | 484 | -3.0625 | Cingulum_Mid_L |
| 6 | -33 | 51 | 232 | -2.9972 | Cingulum_Mid_R |
| 9 | -54 | 9 | 484 | -2.9742 | Lingual_R |
| 6 | -21 | 48 | 232 | -2.9633 | Cingulum_Mid_R |
| -9 | 45 | 18 | 139 | -2.9446 | Cingulum_Ant_L |
| 36 | -18 | 12 | 292 | -2.9052 | Insula_R |
| -51 | -75 | 15 | 255 | -2.8808 | Occipital_Mid_L |
| 51 | -9 | -12 | 292 | -2.8625 | Temporal_Sup_R |
| -3 | 51 | 27 | 139 | -2.8533 | Frontal_Sup_Medial_L |
| 60 | -3 | -12 | 292 | -2.8031 | Temporal_Mid_R |
| -48 | -9 | 3 | 169 | -2.7997 | Temporal_Sup_L |
| 45 | -18 | 0 | 292 | -2.7766 | Temporal_Sup_R |
| -42 | -27 | 21 | 169 | -2.7377 | Rolandic_Oper_L |
| -3 | 36 | 18 | 139 | -2.7372 | Cingulum_Ant_L |
| -6 | -18 | 48 | 232 | -2.7367 | Cingulum_Mid_L |
| 39 | -3 | 9 | 292 | -2.6445 | Insula_R |
| 9 | -72 | 33 | 484 | -2.6094 | Cuneus_R |
| -12 | -60 | 18 | 484 | -2.6061 | Calcarine_L |
| -51 | -21 | 9 | 169 | -2.5862 | Temporal_Sup_L |
| 0 | -60 | 42 | 484 | -2.5068 | Precuneus_L |
| 0 | -45 | 57 | 232 | -2.4941 | Precuneus_L |
| 9 | 36 | 15 | 139 | -2.4886 | Cingulum_Ant_R |
| 18 | -60 | 12 | 484 | -2.3892 | Calcarine_R |
| 12 | -66 | 24 | 484 | -2.3705 | Cuneus_R |
| 3 | -9 | 45 | 232 | -2.2792 | Cingulum_Mid_R |
| 36 | 6 | 9 | 292 | -2.2175 | Insula_R |
| -9 | -51 | 54 | 232 | -2.2101 | Precuneus_L |
| 12 | -45 | 54 | 232 | -2.1233 | Precuneus_R |
| 57 | -24 | 18 | 292 | -2.0851 | Temporal_Sup_R |
