# Supplementary material for: Age-Specific Activation Patterns and Inter-Subject Similarity During Verbal Working Memory Maintenance and Cognitive Reserve
Source: Front Psychol. 2022 Jun 9;13:852995. doi: 10.3389/fpsyg.2022.852995 (PMC9218333; doi:10.3389/fpsyg.2022.852995)
Supplement: Supplementary file 3 [file Table_3.DOCX]

**Supplementary table 3:** 95%iles of the old-minus-young difference distributions.

| MNI-X | MNI-Y | MNI-Z | CS | Fraction | AAL_label |
| --- | --- | --- | --- | --- | --- |
| Old > Young | | | | | |
| -6 | -66 | 60 | 66 | 0.99915 | Precuneus_L |
| 12 | -69 | 57 | 66 | 0.98928 | Precuneus_R |
| 6 | -63 | 63 | 66 | 0.98875 | Precuneus_R |
| Old < Young | | | | | |
| 6 | -21 | 45 | 60 | 0.99594 | Cingulum_Mid_R |
| -6 | -21 | 48 | 60 | 0.99363 | Cingulum_Mid_L |
| -6 | -12 | 48 | 60 | 0.99119 | Cingulum_Mid_L |
| -3 | -30 | 51 | 60 | 0.97168 | Paracentral_Lobule_L |
